# Rural-urban disparities in child nutrition in Bangladesh and Nepal

**DOI:** 10.1186/1471-2458-13-581

**Published:** 2013-06-14

**Authors:** Chittur S Srinivasan, Giacomo Zanello, Bhavani Shankar

**Affiliations:** 1 Department of Food Economics and Marketing, School of Agriculture, Policy and Development, University of Reading, Reading RG6 6AR, UK; 2 Oxford Department of International Development, Queen Elizabeth House, University of Oxford, 3 Mansfield Road, Oxford OX1 3TB, UK; 3 Leverhulme Centre for Integrative Research on Agriculture and Health, School of Oriental and African Studies, University of London, 36, Gordon Square, London WC1H 0XG, UK

**Keywords:** Child nutrition, Socio-economic characteristics, Rural-Urban disparities, Quantile regression, Counterfactual decompositions, Developing countries, Bangladesh, Nepal

## Abstract

**Background:**

The persistence of rural-urban disparities in child nutrition outcomes in developing countries alongside rapid urbanisation and increasing incidence of child malnutrition in urban areas raises an important health policy question - whether fundamentally different nutrition policies and interventions are required in rural and urban areas. Addressing this question requires an enhanced understanding of the main drivers of rural-urban disparities in child nutrition outcomes especially for the vulnerable segments of the population. This study applies recently developed statistical methods to quantify the contribution of different socio-economic determinants to rural-urban differences in child nutrition outcomes in two South Asian countries – Bangladesh and Nepal.

**Methods:**

Using DHS data sets for Bangladesh and Nepal, we apply quantile regression-based counterfactual decomposition methods to quantify the contribution of (1) the differences in levels of socio-economic determinants (covariate effects) and (2) the differences in the strength of association between socio-economic determinants and child nutrition outcomes (co-efficient effects) to the observed rural-urban disparities in child HAZ scores. The methodology employed in the study allows the covariate and coefficient effects to vary across entire distribution of child nutrition outcomes. This is particularly useful in providing specific insights into factors influencing rural-urban disparities at the lower tails of child HAZ score distributions. It also helps assess the importance of individual determinants and how they vary across the distribution of HAZ scores.

**Results:**

There are no fundamental differences in the characteristics that determine child nutrition outcomes in urban and rural areas. Differences in the levels of a limited number of socio-economic characteristics – maternal education, spouse’s education and the wealth index (incorporating household asset ownership and access to drinking water and sanitation) contribute a major share of rural-urban disparities in the lowest quantiles of child nutrition outcomes. Differences in the strength of association between socio-economic characteristics and child nutrition outcomes account for less than a quarter of rural-urban disparities at the lower end of the HAZ score distribution.

**Conclusions:**

Public health interventions aimed at overcoming rural-urban disparities in child nutrition outcomes need to focus principally on bridging gaps in socio-economic endowments of rural and urban households and improving the quality of rural infrastructure. Improving child nutrition outcomes in developing countries does not call for fundamentally different approaches to public health interventions in rural and urban areas.

## Background

Child nutrition outcomes in developing countries have been characterised by large rural-urban disparities over the last few decades [1]. A substantial body of empirical studies shows that average child nutrition outcomes in urban areas are significantly better than in rural areas in a large cross-section of developing countries [2-7]. Van de Poel *et al.*[1] in a study of 47 developing countries show that there are significant differences in rural-urban stunting rates in all but four countries, and that the median rural-urban ratio in stunting is 1.4. The rapid pace of urbanisation in developing countries has at the same time confronted these countries with the growing incidence of child malnutrition and greater nutritional inequalities in urban areas [8]. This persistence of rural-urban disparities in child nutrition alongside growing urbanisation and increasing inequality of child nutrition in urban areas highlights the need for an enhanced understanding of the main drivers of urban-rural differences in nutrition outcomes. An important associated public health policy question is whether fundamentally different nutrition policies and interventions are required in rural and urban areas. For example, in some settings, social support networks are weaker in urban compared to rural areas, and income/wealth may therefore be a more critical constraint for urban compared to rural nutrition outcomes [2]. In such settings, cash transfers may arguably be a more important component of public health/nutrition intervention portfolios in urban areas than in rural ones. Also, the quality of public services of importance to nutrition outcomes, such as education and ante-natal services, have been found severely wanting in some rural areas [9,10]. Such quality differentials could alter the relative effectiveness of key nutrition determinants in rural compared to urban areas, resulting in divergent intervention and policy strategies.

Observed rural-urban differences in indicators of child nutritional outcomes such as Height-for-Age Z scores (HAZ) may arise because of:

(i) rural-urban differences in the *levels* of determinants of nutrition outcomes, such as mother’s education and household wealth – which may be termed as ‘covariate’ effects in a regression context; or

(ii) rural-urban differences in the *strength of association* between particular determinants and nutrition outcomes – which may be termed as coefficient effects in a regression context. For example, an additional year of mother’s education may have a larger impact on nutrition outcomes in an urban or a rural population relative to the other, all else held equal.

Rural-urban disparities in child nutrition may also arise from a combination of covariate and coefficient effects. If rural-urban differences arise largely due to covariate effects, or differing *levels* of determinants, similar policy frameworks and tools could be applied across urban and rural areas [2]. If differences are largely due to coefficient effects, however, strategies may need to vary.

A small literature [2,11] has examined these issues in different settings. We contribute to this literature by applying recently developed statistical methods that allow a more nuanced approach to this ‘covariates or coefficients’ question. These Quantile Regression-based Counterfactual Decomposition (QR-CD) methods allow the covariate and coefficient effects to differ along the entire distribution of nutrition outcomes. For example, are covariate versus coefficient contributions to rural-urban disparities different at the lower tail of the HAZ distribution (where severe stunting is likely to be prevalent) compared to the middle and upper parts of the HAZ distribution? In a policy atmosphere where targeting of the most vulnerable is important, such insights can be valuable. In addition, the methodology we apply also helps assess the importance of individual determinants – *e.g.,* what proportion of rural-urban HAZ score differentials may be explained by differential wealth or maternal education levels, and how does this proportion vary across the HAZ distribution? QR-CD methods are well-validated and have been applied in a variety of regression modelling contexts, including labour remuneration, health outcomes and public finance. There have also been several applications to modelling under as well as over nutrition outcomes in recent years [12-15]. Our application case studies are set in Nepal and Bangladesh, two rapidly urbanising South Asian countries grappling with substantial undernutrition problems. DHS data show that 45% of under-fives in rural Bangladesh are stunted, compared to 36% in urban areas, with a population average of 43%. In Nepal, 51% of rural under-fives are stunted, in comparison to 36% in urban areas, with the population average being 49%. Our primary hypothesis is that most rural-urban disparity across the HAZ distribution arises from covariate, rather than coefficient effects. We are, of course, particularly interested in disparities in the lower tail. A secondary hypothesis is that, even if a covariate or a coefficient effect dominates, there are important differences across the HAZ distribution in the relative contributions of covariate and coefficient effects to rural-urban disparities. If the secondary hypothesis is shown to hold, it would strengthen the rationale for the use in nutrition outcome modelling of approaches considering the entire distribution, such as QR-CD.

### Literature review: determinants of rural-urban disparities and methods used in evaluation

The literature on rural-urban nutrition and health disparities discussed in the previous section has largely modelled the mean/median of nutrition outcomes such as height for age z-scores (HAZ), or the prevalence of stunting or wasting. It has, however, been recognised in the literature that comparisons of means of child nutrition indicators is not adequate for understanding rural-urban disparities. Inequalities of socio-economic endowments tend to be much greater in urban areas. The differing patterns of inequalities in urban and rural areas may imply that rural-urban disparities in the upper and lower tails of the distribution of child nutrition outcomes may be very different from what is suggested by a comparison of means [1,8]. Our interest in this study is not only in studying rural-urban disparities across the entire distribution of nutrition outcomes, but also in examining the influences of specific determinants such as education and wealth on these disparities, and the way in which these influences vary across the distribution of outcomes.

Only a few studies have attempted to quantify the contribution of socio-economic or ecological variables, individually or in the aggregate, to rural-urban differences in child nutrition outcomes. Garrett and Ruel [11] investigated the determinants of the large rural-urban differentials in HAZ outcomes in Mozambique using cross-sectional household survey data in a regression framework modelling mean HAZ. They concluded that the explanation predominantly lay in differing levels of key determinants (covariate effects) rather than differences in the strength of influence of covariates on nutrition outcomes (coefficient effects). Smith et al. [2] examined DHS data from 36 developing countries, again in a (mean) regression framework, and found significant rural-urban differences in the socio-economic and proximate determinants of child nutrition. The study also found very few significant differences in coefficient effects in urban and rural settings and concluded that rural-urban disparities could be predominantly attributed to differences in levels of socio-economic characteristics. Van de Poel et al. [1], using DHS data from 47 developing countries have attempted to quantify the contribution of wealth and other socio-economic characteristics to child nutrition outcomes by examining how rural-urban relative risk ratios for stunting/child mortality change as these characteristics are successively controlled for. They find that on average, rural-urban relative risk ratios fall by 53% when household wealth is controlled for and by a further 23% when other socio economic variables are controlled for. Whilst these studies have provided valuable insights into the determination of urban-rural nutrition outcome differentials, their results only throw light on the mean of the outcome variable. Also, their approaches do not yield the contributions of individual covariates to child nutrition outcomes. We apply QR-CD methods to examine how covariate and coefficient effects, in the aggregate as well as with respect to individual variables, vary throughout the HAZ distribution. Our primary hypothesis, that most rural-urban disparity across the HAZ distribution arises from covariate, rather than coefficient effects, essentially tests that the main insight available from the previous literature modelling the mean of HAZ extends to the entire HAZ distribution, and in particular, the lower tail.

### Data and variables

For the empirical application of this approach, we have chosen two country case studies in South Asia – Bangladesh and Nepal. Both countries are developing countries with a high incidence of poverty-31.5% in Bangladesh (2010) and 25.2% in Nepal (2011) [16] - and significant rural-urban disparities in child nutrition – but differ substantially in the extent of urbanisation. While only 17% of Nepal’s population lives in urban areas, nearly 33% of the population of Bangladesh is urban, making it one of the more urbanised countries in South Asia [17]. The two countries also differ significantly in levels of maternal education and child vaccination coverage (Table 1). The two case studies allow the examination of rural-urban differences when key socio-economic determinants and the extent of urbanisation are substantially different. We do not pool data from the two countries. Instead we treat them as distinct case studies and provide separate estimates for each, although we provide a broad comparative discussion of results.

**Table 1:** Child nutrition and socio-economic characteristics in Bangladesh and Nepal

	**Bangladesh**			
	**Aggregate (Urban + Rural) Number of children = 5267**	**Urban (Number of children = 1842)**	**Rural (Number of children = 3425)**	**Rural Urban difference**^**a**^
	**Mean (Std deviation in brackets)**	**Mean (Std deviation in brackets)**	**Mean (Std deviation in brackets)**	
Height/Age (z-score)	−1.72 (1.36)	−1.49 (1.35)	−1.84 (1.35)	0.35***
Gender of Child (female = 1)	0.50 (0.50)	0.47 (0.50)	0.51 (0.50)	−0.034*
Age of Child	1.99 (1.41)	1.94 (1.38)	2.01 (1.42)	−0.07
Child Vaccinated (yes = 1)	0.49 (0.50)	0.52 (0.50)	0.48 (0.50)	0.043**
Age of mother	25.86 (6.17)	26.00 (5.94)	25.78 (6.29)	0.22
Mother currently working (yes = 1)	0.24 (0.42)	0.24 (0.43)	0.23 (0.42)	0.005
Years of education mother	4.91 (4.33)	5.94 (4.69)	4.35 (4.02)	1.60***
Years of education of spouse	4.88 (4.88)	6.21 (5.22)	4.16 (4.53)	2.04***
Wealth status (index)	−0.09 (0.93)	0.55 (1.14)	−0.41 (0.58)	1.0***
Extended family dummy (yes = 1)	0.44 (0.50)	0.44 (0.50)	0.44 (0.50)	0.00
Dependency ratio	1.12 (0.72)	0.99 (0.65)	1.19 (0.75)	−0.19***
Number of children <5 yrs	1.39 (0.57)	1.34 (0.55)	1.42 (0.58)	−0.07***
	**Nepal**			
	**Aggregate (Urban + Rural) Number of children = 5219**	**Urban (Number of children = 1168)**	**Rural (Number of children = 4051**	**Rural Urban difference**^a^
	**Mean (Std deviation in brackets)**	**Mean (Std deviation in brackets)**	**Mean (Std deviation in brackets)**	
Height/Age (z-score)	−1.96 (1.34)	−1.65 (1.36)	−2.05 (1.31)	0.4***
Gender Child (female = 1)	0.49 (0.50)	0.50 (0.50)	0.49 (0.50)	0.01
Age of child	2.05 (1.40)	2.11 (1.43)	2.03 (1.39)	0.08
Child Vaccinated (yes = 1)	0.24 (0.42)	0.26 (0.44)	0.23 (0.42)	0.031*
Age of mother	26.96 (6.07)	26.18 (5.27)	27.18 (6.26)	−1.00***
Mother currently working (yes = 1)	0.70 (0.46)	0.54 (0.50)	0.75 (0.43)	−0.21***
Years of education mother	2.46 (3.65)	4.21 (4.31)	1.95 (3.27)	2.26***
Years of education of spouse	5.28 (4.09)	6.76 (4.34)	4.85 (3.91)	1.91***
Wealth status (index)	−0.21 (0.84)	0.52 (1.16)	−0.43 (0.54)	0.96***
Extended family dummy (yes = 1)	0.51 (0.50)	0.47 (0.50)	0.52 (0.50)	−0.05**
Dependency ratio	1.38 (0.96)	1.18 (0.81)	1.44 (0.99)	−0.26***
Number of children < 5 years	1.58 (0.63)	1.50 (0.63)	1.61 (0.62)	−0.10***

^a^ Asterisk in the column indicate the level of significance of the difference in outcomes/characteristics between rural and urban areas based on independent sample T-tests. *** denotes significance at 1% level, ** denotes significance at 5% level and * denotes significance at the 10% level of significance.

We have used datasets from the Demographic and Health Surveys of the MEASURE-DHS project (http://measuredhs.com/) which collects and disseminates nationally representative demographic, health and nutrition information based on household surveys for 90 countries. The datasets are freely accessible to the public and researchers subject to a prescribed registration and approval process. Permission to access and use the datasets relevant to this study was obtained by the authors from the MEASURE-DHS archive. The most recent datasets from the Demographic and Health Surveys for Bangladesh (2007) and Nepal (2006) were used in the study. The datasets include data from a nationally representative sample of urban and rural households. Units for observation for this study were all children aged below five years in the households surveyed. After deletion of observations with incomplete information, the sample for Bangladesh had 5267 children, with 1842 (35%) living in urban households and 3425 (65%) living in rural households, while Nepal had 5219 children with 1168 (22%) living in urban households 4051 (78%) living in rural households.

We used height-for-age Z-scores (HAZ) as indicators of child nutrition in rural and urban households. Stunting, defined as HAZ less than two standard deviations of the NCHS/CDC/WHO International Reference Standard [18], is a good indicator of child nutrition and health status as it reflects the effects of chronic nutritional deficiency. Determinants of child nutrition status used in this study are mostly based on the previous literature and include child characteristics as well as socio-economic characteristics of the household. Gender, age of the child and child vaccination are the child characteristics included in this study. Socio-economic characteristics of the household included in this study are years of education of the mother and the spouse, employment status of the mother (whether the mother is currently working), dependency ratio (computed as the ratio of economically inactive members of the household (under 16 and over 64 years old) to active members, the number of children below five years in the household, whether the household is an extended family unit and an indicator of socio-economic status – the DHS wealth index.

The DHS wealth index [19] is a composite measure of a household’s relative economic status and has been extensively used in the assessment of equity in health services and distribution of services among the poor [20-26]. In environments where accurate data on income and expenditure are extremely difficult to collect or may be subject to considerable volatility, the DHS wealth index provides a more stable and reliable measure of a household’s cumulative living standard and access to utilities and health care. The indicator variables used for construction of the DHS Wealth Index include household assets and utility services recorded in the DHS surveys ^a^. For the construction of the Index, these variables are broken into sets of dichotomous variables and indicator weights are assigned using principal component analysis (PCA) as suggested by Filmer and Pritchett [27]. The indicator variables are first standardised (z-scores are calculated) and then factor coefficient (factor loading) scores are calculated. For each household the indicator values are multiplied by the factor loadings to produce the household’s index value. The index value itself is a standardised score with a mean of zero and a standard deviation of one ^b^.

## Methods

### Conceptual framework

The conceptual framework underpinning our empirical analysis is the widely-applied UNICEF framework [28] outlining the causes of undernutrition. In the UNICEF framework, child malnutrition can be analysed in terms of immediate, underlying and basic causes. The immediate causes are inadequate dietary intakes and infectious disease, the underlying causes are inadequate maternal and child care, inadequate health services and health environment and the basic causes are institutional and socio-economic determinants and potential resources. The basic causes can be viewed as “exogenous” determinants – which influence child nutrition through their effect on the intervening proximate determinants. The proximate determinants are, therefore, endogenously determined by the exogenous characteristics. In empirical (reduced form) models examining the relationship between child nutrition outcomes and exogenous characteristics, the proximate determinants will generally be excluded to prevent biased and uninterpretable parameters [2,29].

### Statistical methods

To assess the rural-urban differentials in HAZ scores, we first estimate the distributions of HAZ scores separately for rural and urban children in each country using kernel smoothing techniques. From the kernel density estimates of HAZ scores, the rural-urban differential is computed at each quantile and provides the raw difference in HAZ scores across the distribution.

A major objective of this study is to decompose the rural-urban differences in child nutrition outcomes into the covariate (or composition) effect, i.e., the differences in HAZ scores due to differences in levels of characteristics of urban and rural households, and the co-efficient (or structure) effect, i.e., the differences in HAZ scores due to the differences in the returns to those characteristics, across the entire distribution of HAZ scores. Linear or logistic regression approaches assess the mean response of the outcome variable to changes in covariates and the effect of covariates is constrained to be the same along the entire distribution of the outcome variable. Decompositions based on linear regression results [30,31] would apply only to the mean rural-urban differences in HAZ scores, but not to other distributional statistics like quantiles. We, therefore, use a quantile regression (QR) approach to assess how child nutrition outcomes are related to individual and household characteristics. The QR technique allows the impact of explanatory variables to vary along the entire distribution of the outcome variable – HAZ scores in our case. The QR method allows us to understand how the effects of covariates in the lowest quantile of HAZ scores may differ from those in other quantiles. For instance, the impact of an increase in mother’s education may be very different in the higher and lower tails of HAZ scores. Koenker and Hallock [32] warn against the temptation to simply segment the outcome variable, *e.g.,* HAZ, into subsets based on outcomes values, *e.g.,* deciles of HAZ values, and run standard regressions on these segments separately, since this introduces sample selectivity problems. Estimating categorical dependent variables models is one option, *e.g.,* probit or logit models to explain stunting status. However, apart from constraining the effect of explanatory covariates to be the same across the distribution of outcomes, these models sacrifice statistical information in grouping continuously distributed variables like HAZ into small numbers of categories. QR methods offer the most robust approach to flexibly model the shifts in HAZ distribution associated with changes to covariates.

The quantile regression method developed by Koenker and Bassett [33] estimates only the conditional quantile effects of changes in explanatory variables. In assessing the impact of policy interventions or understanding the impacts of transitions such as urbanisation, we are more interested in the effect of a change in an explanatory variable (e.g., years of education of mothers) in a population of individuals with different characteristics (unconditional effects) rather than in the impact for sub-groups with specific values of covariates (conditional effects). To assess the unconditional quantile effects of changes in explanatory variables, we use an unconditional Recentred Influence Function (RIF) QR regression method developed by Firpo et al. [34]. A linear specification was adopted for the unconditional QR. However, we did test for the presence of non-linear associations between parental education (mother’s education and spouse’s education) and HAZ scores by introducing a quadratic term and for threshold effects by introducing dummy variables for different levels of education (no education, primary education, secondary education). We found no statistically significant evidence of non-linear associations or threshold effects for parental education and the additional variables did not affect the magnitude or significance of other explanatory variables. This supported the adoption of a linear specification for the QR.

Following Firpo et al. [34], the decomposition of differences between rural and urban HAZ scores (for each country) proceeds in two steps (please see Additional file 1 for details of the decomposition procedure). In the first step, a counterfactual distribution of urban HAZ scores is constructed [35] which is the distribution of HAZ scores in urban areas that would have prevailed if urban households had the same returns to their characteristics as the rural population. The difference between the distribution of the rural HAZ scores and the counterfactual distribution gives the covariate effect and the difference between the counterfactual distribution and the distribution of urban HAZ scores gives the coefficient effect. The covariate and coefficient effects are each decomposed into the contribution of individual covariates using the Recentred Influence Function (RIF) regression to obtain unconditional quantile effects of covariates on HAZ scores [36,37].

Although our set of chosen covariates excludes proximate determinants in order to minimize endogeneity problems, and is consistent with variables used in previous literature [2,11,13], there is still potential for lingering endogeneity leading to difficulties in parameter interpretation. The education variables in our models are a case in point. Parental education may simply be correlated with unobserved parental values and skills that influence child height, complicating any causal attribution. However, it is important to make clear, as O’Donnell *et al.*[13] note, that the objective of counterfactual decomposition is not causal identification, but rather to explain variations in child height and judge the relative values of covariate and coefficient effects. Caution is warranted in the interpretation of coefficients of variables that are potentially endogenous, but the decomposition itself remains valid.

## Results

### Descriptive statistics

Table 1 provides descriptive statistics of child nutrition outcomes and characteristics in rural and urban areas within Bangladesh and Nepal. In both Bangladesh and Nepal, rural households have significantly worse HAZ scores than urban areas. The difference in mean HAZ scores between urban and rural households is 0.35 in Bangladesh, while it is 0.40 in Nepal. In both countries, urban mothers and spouses have more education than their rural counterparts. Also urban households in both countries are wealthier (as measured by the DHS wealth index), have fewer children under the age of 5 years, a higher proportion of vaccinated children, a lower dependency ratio and lower likelihood of living in extended families. Differences in urban and rural outcomes and characteristics are all statistically significant in both countries (except for the age of the child in both countries and the proportion of male and female children in Nepal).

### Quantile regression results

The estimates of the unconditional RIF quantile regressions (QR) separately for rural and urban areas are shown for Bangladesh and Nepal in Tables 2 and 3 respectively. In Bangladesh, only child’s age, mother’s education, spouse’s education and the wealth index are seen to have a consistently significant association with HAZ scores across the HAZ distribution in both rural and urban areas. Increases in child age tend to lower HAZ scores, reflecting growth faltering in young children in the region. This effect increases substantially as we move from the lower tail to the upper tail in both rural and urban areas, indicating that children starting with better nutritional status stand to lose more through faltering as they grow older. This pattern underlines the importance of flexibly modelling effects across the distribution. Better education of the mother is associated with improved nutrition, as expected. In rural areas, this effect is particularly important for the most undernourished, with the effect wearing off in the upper half of the HAZ distribution. In urban areas, this relationship remains relatively stable throughout the distribution. Higher spousal education and wealth both display a positive relationship with HAZ scores in both rural and urban areas. The spouse education-HAZ relationship remains broadly similar throughout the distributions, while the wealth index-HAZ relationship gets stronger in the upper part of the distribution for rural areas.

**Table 2:** Unconditional Recentred Influence Function (RIF) quantile regression results for rural and urban households in Bangladesh

**Dependent variable: HAZ score**												
	**RURAL**						**URBAN**					
	**OLS**	**Quantiles**					**OLS**	**Quantiles**				
		**10**	**25**	**50**	**75**	**90**		**10**	**25**	**50**	**75**	**90**
Female gender of child	−0.04	−0.01	0.05	−0.05	−0.08	−0.27***	0.04	0.00	0.03	0.08	0.08	−0.08
	(0.04)	(0.08)	(0.06)	(0.05)	(0.06)	(0.09)	(0.06)	(0.10)	(0.08)	(0.08)	(0.08)	(0.10)
Age of Child	−0.24***	−0.11***	−0.15***	−0.23***	−0.31***	−0.44***	−0.20***	−0.08**	−0.14***	−0.19***	−0.26***	−0.28***
	(0.02)	(0.03)	(0.02)	(0.02)	(0.03)	(0.04)	(0.02)	(0.03)	(0.03)	(0.03)	(0.04)	(0.05)
Child vaccinated	−0.18***	0.08	0.01	−0.20***	−0.33***	−0.41***	−0.06	0.05	−0.04	−0.09	−0.12	−0.09
	(0.05)	(0.08)	(0.06)	(0.06)	(0.06)	(0.09)	(0.06)	(0.10)	(0.09)	(0.07)	(0.08)	(0.11)
Age of mother	0.00	−0.02**	−0.01	0.00	0.00	0.01*	0.02***	−0.01	0.00	0.01	0.03***	0.03***
	(0.00)	(0.01)	(0.01)	(0.00)	(0.01)	(0.01)	(0.01)	(0.01)	(0.01)	(0.01)	(0.01)	(0.01)
Mother’s working status	0.09*	0.13	0.13*	0.09	0.10	0.13	−0.04	−0.20	−0.03	−0.05	0.04	0.03
	(0.05)	(0.10)	(0.07)	(0.07)	(0.07)	(0.10)	(0.07)	(0.14)	(0.11)	(0.09)	(0.09)	(0.12)
Mother’s education (yrs)	0.01*	0.04**	0.02**	0.01*	0.02	0.00	0.04***	0.03**	0.04***	0.05***	0.04**	0.04**
	(0.01)	(0.01)	(0.01)	(0.01)	(0.01)	(0.02)	(0.01)	(0.02)	(0.01)	(0.01)	(0.01)	(0.02)
Husband’s education (yrs)	0.02***	0.02**	0.02***	0.02***	0.02***	0.03**	0.04***	0.04**	0.05***	0.04***	0.05***	0.03**
	(0.01)	(0.01)	(0.01)	(0.01)	(0.01)	(0.01)	(0.01)	(0.02)	(0.01)	(0.01)	(0.01)	(0.01)
Wealth index	0.26***	0.20***	0.27***	0.29***	0.27***	0.37***	0.13***	0.09	0.08*	0.16***	0.11**	0.13**
	(0.04)	(0.07)	(0.06)	(0.06)	(0.06)	(0.11)	(0.03)	(0.06)	(0.05)	(0.04)	(0.05)	(0.06)
Extended family dummy	0.05	−0.12	−0.02	0.04	0.05	0.22**	0.07	−0.01	0.01	0.12	0.14	0.05
	(0.05)	(0.09)	(0.07)	(0.06)	(0.07)	(0.09)	(0.06)	(0.10)	(0.08)	(0.08)	(0.09)	(0.11)
Dependency ratio	−0.05	−0.08	−0.04	−0.10**	−0.07	−0.05	−0.02	0.01	0.05	−0.08	−0.04	0.06
	(0.03)	(0.06)	(0.05)	(0.04)	(0.04)	(0.06)	(0.05)	(0.09)	(0.07)	(0.06)	(0.06)	(0.09)
No. of children (<5 yrs)	0.02	−0.07	−0.04	0.06	0.11**	0.12	0.03	0.02	−0.05	0.00	0.10	0.09
	(0.04)	(0.08)	(0.06)	(0.05)	(0.05)	(0.08)	(0.06)	(0.10)	(0.09)	(0.07)	(0.07)	(0.09)
Constant	−1.25***	−2.76***	−2.20***	−1.30***	−0.47**	0.50*	−2.10***	−3.37***	−2.65***	−2.02***	−1.50***	−0.74**
	(0.14)	(0.30)	(0.20)	(0.16)	(0.19)	(0.27)	(0.18)	(0.31)	(0.25)	(0.24)	(0.24)	(0.32)
N	3425	3425	3425	3425	3425	3425	1842	1842	1842	1842	1842	1842
R-sq	0.11	0.03	0.05	0.08	0.09	0.07	0.18	0.04	0.09	0.15	0.13	0.07
adj. R-sq	0.10	0.03	0.05	0.07	0.08	0.07	0.18	0.04	0.09	0.15	0.12	0.07

Figures in brackets are standard errors: Asterisks denote level of significance -***, ** and * denote significance at 1%, 5% and 10% level of significance respectively.

**Table 3:** Unconditional Recentred Influence Function (RIF) quantile regression results for rural and urban households in Nepal

**Dependent variable: HAZ score**												
	**RURAL**						**URBAN**					
	**OLS**	**Quantiles**					**OLS**	**Quantiles**				
		**10**	**25**	**50**	**75**	**90**		**10**	**25**	**50**	**75**	**90**
Female gender of child	−0.01	−0.03	−0.06	0.02	0.02	0.09	0.02	0.08	0.01	0.02	0.01	−0.09
	(0.04)	(0.06)	(0.05)	(0.05)	(0.06)	(0.08)	(0.07)	(0.12)	(0.11)	(0.08)	(0.10)	(0.14)
Age of child	−0.23***	−0.09***	−0.12***	−0.22***	−0.31***	−0.41***	−0.27***	−0.23***	−0.24***	−0.29***	−0.26***	−0.30***
	(0.02)	(0.03)	(0.02)	(0.02)	(0.03)	(0.04)	(0.03)	(0.04)	(0.04)	(0.04)	(0.04)	(0.06)
Child vaccinated	0.21***	0.16**	0.13**	0.20***	0.29***	0.39***	0.29***	−0.03	0.19*	0.24**	0.45***	0.62***
	(0.05)	(0.08)	(0.06)	(0.06)	(0.08)	(0.12)	(0.09)	(0.14)	(0.11)	(0.11)	(0.13)	(0.20)
Age of mother	0.00	−0.01	−0.01**	−0.01	0.00	0.01	0.01	−0.02	0.00	0.02**	0.01	0.01
	(0.00)	(0.01)	(0.01)	(0.00)	(0.01)	(0.01)	(0.01)	(0.01)	(0.01)	(0.01)	(0.01)	(0.02)
Mother’s working status	−0.17***	−0.11	−0.14***	−0.12**	−0.14**	−0.29***	−0.12	−0.12	−0.10	0.03	−0.22**	−0.23
	(0.05)	(0.07)	(0.05)	(0.06)	(0.07)	(0.11)	(0.08)	(0.14)	(0.11)	(0.10)	(0.11)	(0.17)
Mother’s education (yrs)	0.05***	0.03**	0.04***	0.05***	0.05***	0.08***	0.06***	0.04*	0.06***	0.07***	0.06***	0.06**
	(0.01)	(0.01)	(0.01)	(0.01)	(0.01)	(0.02)	(0.01)	(0.02)	(0.02)	(0.01)	(0.02)	(0.02)
Husband’s education (yrs)	0.01**	0.03***	0.02*	0.00	0.01	0.01	0.00	0.01	0.03*	0.01	−0.02	−0.01
	(0.01)	(0.01)	(0.01)	(0.01)	(0.01)	(0.01)	(0.01)	(0.02)	(0.02)	(0.01)	(0.02)	(0.02)
Wealth index	0.22***	0.17***	0.22***	0.31***	0.30***	0.16	0.15***	0.10	0.05	0.15***	0.24***	0.16
	(0.04)	(0.05)	(0.04)	(0.05)	(0.07)	(0.11)	(0.04)	(0.07)	(0.05)	(0.05)	(0.06)	(0.10)
Extended family dummy	−0.01	0.04	0.02	−0.02	−0.05	−0.04	−0.01	−0.06	−0.14	−0.11	0.04	−0.02
	(0.04)	(0.07)	(0.05)	(0.05)	(0.06)	(0.09)	(0.07)	(0.13)	(0.10)	(0.09)	(0.10)	(0.15)
Dependency ratio	−0.06**	−0.05	−0.02	−0.07***	−0.05	−0.08*	−0.04	−0.14	−0.14*	−0.06	0.05	0.05
	(0.02)	(0.05)	(0.03)	(0.03)	(0.03)	(0.04)	(0.05)	(0.10)	(0.07)	(0.05)	(0.06)	(0.09)
No. of children(<5 yrs)	−0.03	−0.06	−0.08*	−0.04	0.03	0.08	−0.05	−0.09	−0.04	0.00	−0.04	−0.02
	(0.03)	(0.06)	(0.04)	(0.04)	(0.04)	(0.07)	(0.06)	(0.13)	(0.09)	(0.07)	(0.07)	(0.11)
Constant	−1.32***	−3.11***	−2.22***	−1.29***	−0.44***	0.24	−1.44***	−2.33***	−2.20***	−2.00***	−0.76**	−0.01
	(0.13)	(0.22)	(0.18)	(0.17)	(0.19)	(0.28)	(0.24)	(0.48)	(0.32)	(0.29)	(0.34)	(0.46)
N	4051	4051	4051	4051	4051	4051	1168	1168	1168	1168	1168	1168
R-sq	0.15	0.03	0.06	0.11	0.12	0.09	0.23	0.07	0.12	0.18	0.16	0.09
adj. R-sq	0.15	0.03	0.06	0.11	0.11	0.08	0.22	0.06	0.12	0.17	0.16	0.08

Figures in brackets are standard errors: Asterisks denote level of significance -***, ** and * denote significance at 1%, 5% and 10% level of significance respectively.

The distribution-wide relationship between age of the child and HAZ scores in rural and urban areas in Nepal is similar to that of Bangladesh. Again, mother’s education has a consistently positive relationship with HAZ scores across the distribution in both rural and urban areas. There are two key differences between the Nepal and Bangladesh results. In contrast to Bangladesh, in Nepal, education of the spouse has only a weak and largely insignificant relationship with child nutrition. However, child vaccination has a positive and significant association with HAZ scores. In both rural and urban areas, this relationship strengthens as we move up the HAZ score distribution.

### Counterfactual decompositions

**Figure 1:**
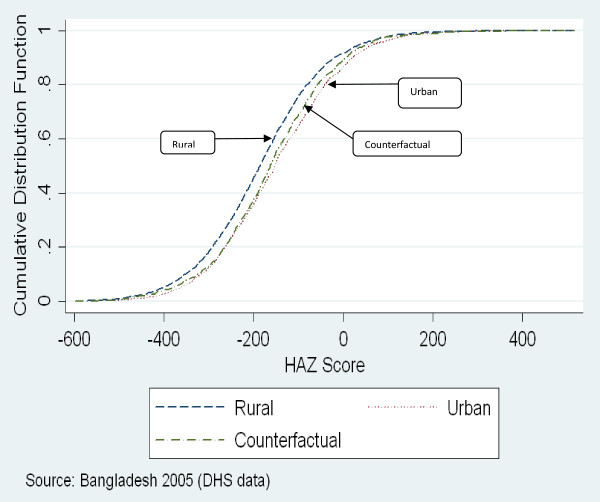
Distribution of Rural and Urban HAZ scores in Bangladesh.

**Figure 2:**
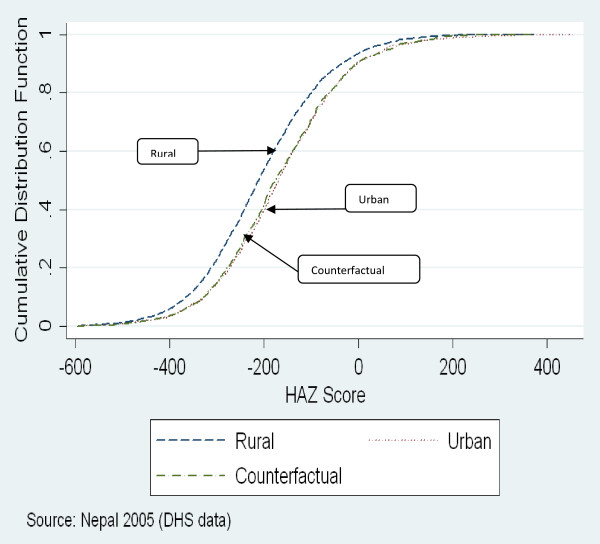
Distribution of Rural and Urban HAZ scores in Nepal.

Figures 1 and 2 show the cumulative distribution functions for urban and rural HAZ scores in Bangladesh and Nepal, respectively. They also depict in aggregate the results of the QR-CD analysis. The curves marked ‘counterfactual’ in the two figures depict the distribution of urban HAZ scores that would prevail if urban households had the same returns to their characteristics (covariates) as rural households. The differences in rural and urban HAZ scores across quantiles, the decomposition of these differences into aggregate covariate and co-efficient effects and the contribution of individual characteristics to these effects are presented in Table 4 for Bangladesh and in Table 5 for Nepal.

**Table 4:** Decomposition of rural-urban differences in child nutrition outcomes – Bangladesh

	**Quantiles**										
	**Q10**		**Q25**		**Q50**			**Q75**		**Q90**	
Rural HAZ scores	−3.5188		−2.7013		−1.8577			−1.0179		−0.1760	
Urban HAZ scores	−3.1753		−2.4321		−1.5227			−0.6166		0.2152	
Observed raw gap in HAZ scores^a^	−0.3436***		−0.2692***		−0.3350***			−0.4013***		−0.3912***	
Covariate effect (% contribution)	−0.2694*** *(78.43%)*		−0.2555*** *(94.95%)*		−0.2493*** *(74.39%)*			−0.2816*** *(70.17%)*		−0.2434*** *(62.19%)*	
Co-efficient effect (% contribution)	−0.0741 *(21.57%)*		−0.0136 *(5.05%)*		−0.0858 *(25.58%)*			−0.1197 *(29.83%)*		−0.1478* *(37.78%)*	
	**Contribution of individual characteristics to rural-urban differences in HAZ scores**^**b**^										
	**Covariate effect**						**Co-efficient effect**				
	**Q10**	**Q25**	**Q50**	**Q75**	**Q90**		**Q10**	**Q25**	**Q50**	**Q75**	**Q90**
Size of effect	−0.2694***	−0.2555***	−0.2493***	−0.2816***	−0.2434***		−0.0741	−0.0136	−0.0858	−0.1197	−0.1478*
**Explained**											
Female gender of child	−0.0001 (0.05%)	0.0003 (-0.15%)	−0.0002 (0.11%)	−0.0004 (0.22%)	−0.0014 (0.67%)		−0.0001 (0.14%)	0.0010 (-1.42%)	0.0024 (-2.20%)	0.0023 (-2.78%)	−0.0022 (2.09%)
Age of child	−0.0060* (3.16%)	−0.0085* (4.38%)	−0.0127* (6.69%)	−0.0171* (9.59%)	−0.0246* (11.76%)		−0.0014 (2.00%)	−0.0026 (3.69%)	−0.0035 (3.21%)	−0.0048 (5.81%)	−0.0051 (4.84%)
Child vaccinated	−0.0039 (2.06%)	−0.0004 (0.21%)	0.0095*** (-5.00%)	0.0161*** (-9.03%)	0.0199*** (-9.52%)		0.0003 (-0.43%)	−0.0002 (0.28%)	−0.0005 (0.46%)	−0.0007 (0.85%)	−0.0005 (0.47%)
Age of mother	−0.0021 (1.11%)	−0.0009 (0.46%)	0.0000 (0.00%)	0.0004 (-0.22%)	0.0015 (-0.72%)		0.0016 (-2.29%)	0.0011 (-1.56%)	−0.0035 (3.21%)	−0.0080 (9.69%)	−0.0106 (10.07%)
Mother’s working status	−0.0027 (1.42%)	−0.0027 (1.39%)	−0.0018 (0.95%)	−0.0021 (1.18%)	−0.0026 (1.24%)		−0.0030 (4.29%)	−0.0004 (0.57%)	−0.0007 (0.64%)	0.0006 (-0.73%)	0.0005 (-0.47%)
Mother’s education (yrs)	−0.0495*** (26.09%)	−0.0312*** (16.08%)	−0.0194** (10.22%)	−0.0214** (12.00%)	0.0065 (-3.11%)		−0.0066 (9.43%)	−0.0094 (13.33%)	−0.0096 (8.81%)	−0.0076 (9.20%)	−0.0087 (8.26%)
Spouse’s education (yrs)	−0.0424*** (22.35%)	−0.0394*** (20.31%)	−0.0339*** (17.85%)	−0.0423*** (23.72%)	−0.0453*** (21.66%)		−0.0103 (14.71%)	−0.0127 (18.01%)	−0.0116 (10.64%)	−0.0141 (17.07%)	−0.0078 (7.41%)
Wealth index	−0.0748*** (39.43%)	−0.1026*** (52.89%)	−0.1122*** (59.08%)	−0.1019*** (57.15%)	−0.1404*** (67.14%)		−0.0497** (71.00%)	−0.0482*** (68.37%)	−0.0948*** (86.97%)	−0.0643*** (77.85%)	−0.0757*** (71.89%)
Extended family dummy	0.0122* (-6.43%)	0.0023 (-1.19%)	−0.004 (2.11%)	−0.0047 (2.64%)	−0.0225*** (10.76%)		−0.0008 (1.14%)	0.0011 (-1.56%)	0.0123** (-11.28%)	0.0140** (-16.95%)	0.0055 (-5.22%)
Dependency ratio	−0.0151 (7.96%)	−0.0079 (4.07%)	−0.0196*** (10.32%)	−0.0130** (7.29%)	−0.0098 (4.69%)		−0.0001 (0.14%)	−0.0003 (0.43%)	0.0005 (-0.46%)	0.0003 (-0.36%)	−0.0004 (0.38%)
No. of children < 5 yrs	−0.0051 (2.69%)	−0.0031 (1.60%)	0.0043 (-2.26%)	0.0082** (-4.60%)	0.0097** (-4.64%)		−0.0001 (0.14%)	0.0002 (-0.28%)	0.0000 (0.00%)	−0.0003 (0.36%)	−0.0003 (0.28%)
Total	−0.1897*** (100%)	−0.1940*** (100%)	−0.1899*** (100%)	−0.1783*** (100%)	−0.2091*** (100%)		−0.0700*** (100%)	−0.0705** (100%)	−0.1090*** (100%)	−0.0826*** (100%)	−0.1053*** (100%)
**Unexplained**											
Residual	−0.0797	−0.0615	−0.0593	−0.1033*	−0.0343		−0.0041	0.0569	0.0233	−0.0371	−0.0426

^a^Raw gap in HAZ scores computed as rural HAZ scores- urban HAZ scores. The negative sign of the observed raw gap figures reflects the fact that rural HAZ scores are lower than urban HAZ scores.

^b^Figures in brackets show percentage contribution of individual characteristics to the total explained effect.

Significant effects are in bold. Asterisks denote level of significance - ***, ** and * denote significance at the 1%, 5% and 10% level of significance respectively.

**Table 5:** Decomposition of rural-urban differences in child nutrition outcomes – Nepal

	**Quantiles**										
	**Q10**		**Q25**		**Q50**			**Q75**		**Q90**	
Rural HAZ scores	−3.6498***		−2.9145***		−2.1065***			−1.2287***		−0.3370***	
Urban HAZ scores	−3.2651***		−2.5150***		−1.6845***			−0.8250***		−0.0186	
Observed raw gap in HAZ scores^a^	−0.3847***		−0.3995***		−0.4220***			−0.4037***		−0.3184***	
Covariate effect (% contribution)	−0.3744*** (97.32%)		−0.3484*** (87.21%)		−0.3045*** (72.16%)			−0.3404*** (84.32%)		−0.2608*** (81.91%)	
Co-efficient effect (% contribution)	−0.0103 (2.68%)		−0.0510 (12.77%)		−0.1175* (27.87%)			−0.0633 (15.68%)		−0.0576 (18.09%)	
	**Contribution of individual characteristics to rural-urban differences in HAZ scores**^**b**^										
	**Covariate effect**						**Co-efficient effect**				
	**Q10**	**Q25**	**Q50**	**Q75**	**Q90**		**Q10**	**Q25**	**Q50**	**Q75**	**Q90**
Size of effect	−0.3744***	−0.3484***	−0.3045***	−0.3404***	−0.2608***		−0.0103	−0.0510	−0.1175*	−0.0633	−0.0103
**Explained**											
Female gender of child	0.0001 (-0.04%)	0.0001 (-0.03%)	0.0000 (0.00%)	0.0000 (0.00%)	−0.0002 (0.06%)		−0.0001 (0.24%)	0.0000 (0.00%)	0.0000 (0.00%)	0.0000 (0.00%)	0.0001 (-0.15%)
Age of child	0.0043 (-1.71%)	0.0057 (-1.99%)	0.0102 (-3.06%)	0.0143 (-4.41%)	0.0189 (-5.66%)		0.0090 (-21.18%)	0.0092 (-31.51%)	0.0112 (-18.54%)	(0.0101 (-10.10%)	0.0118 (-17.35%)
Child vaccinated	−0.0032 (1.27%)	−0.0026 (0.91%)	−0.0040* (1.20%)	−0.0059* (1.82%)	−0.0080* (2.40%)		0.0003 (-0.71%)	−0.0020 (6.85%)	−0.0025 (4.14%)	−0.0047 (4.70%)	−0.0064 (9.41%)
Age of mother	−0.0102** (4.05%)	−0.0106*** (3.70%)	−0.0054* (1.62%)	−0.0041 (1.26%)	0.0085 (-2.54%)		0.0010 (-2.35%)	0.0000 (0.00%)	−0.0011 (1.82%)	−0.0004 (0.40%)	−0.0008 (1.18%)
Mother’s working status	−0.0210** (8.33%)	−0.0258*** (9.00%)	−0.0224*** (6.71%)	−0.0267*** (8.23%)	−0.0538*** (16.11%)		−0.0028 (6.59%)	−0.0023 (7.88%)	0.0007 (-1.16%)	−0.0054 (5.40%)	−0.0054 (7.94%)
Mother’s education (yrs)	−0.0560*** (22.21%)	−0.0944*** (32.93%)	−0.1174*** (35.17%)	−0.1091*** (33.63%)	−0.1794*** (53.71%)		−0.0016 (3.76%)	−0.0022 (7.53%)	−0.0029 (4.80%)	−0.0025 (2.50%)	−0.0022 (3.24%)
Spouse’s education (yrs)	−0.0554*** (21.98%)	−0.0290*** (10.12%)	−0.0036 (1.08%)	−0.0204* (6.29%)	−0.0177 (5.30%)		−0.0013 (3.06%)	−0.0026 (8.90%)	−0.0014 (2.32%)	0.0016 (-1.60%)	0.0007 (-1.03%)
Wealth index	−0.0950*** (37.68%)	−0.1197*** (41.75%)	−0.1704*** (51.05%)	−0.1626*** (50.12%)	−0.0880** (26.35%)		−0.0409** (96.24%)	−0.0218* (74.66%)	−0.0600*** (99.34%)	−0.0998*** (99.80%)	−0.0655*** (96.32%)
Extended family dummy	0.0007 (-0.28%)	0.0004 (-0.14%)	−0.0003 (0.09%)	−0.0009 (0.28%)	−0.0007 (0.21%)		−0.0023 (5.41%)	−0.0049 (16.78%)	−0.0037 (6.13%)	0.0013 (-1.30%)	−0.0006 (0.88%)
Dependency ratio	−0.0114 (4.52%)	−0.0046 (1.60%)	−0.0176*** (5.27%)	−0.0114** (3.51%)	−0.0202*** (6.05%)		−0.0018 (4.24%)	−0.0017 (5.82%)	−0.0008 (1.32%)	0.0006 (-0.60%)	0.0006 (-0.88%)
No. of children < 5 yrs	−0.0049 (1.94%)	−0.0061** (2.13%)	−0.0029 (0.87%)	0.0026 (-0.80%)	0.0065 (-1.95%)		−0.0020 (4.71%)	−0.0009 (3.08%)	0.0001 (-0.17%)	−0.0008 (0.80%)	−0.0004 (0.59%)
Total	−0.2521*** (100%)	−0.2867*** (100%)	−0.3338*** (100%)	−0.3244*** (100%)	−0.3340*** (100%)		−0.0425 (100%)	−0.0292 (100%)	−0.0604* (100%)	−0.1000*** (100%)	−0.0680* (100%)
**Unexplained**											
Residual	−0.0571	−0.0617	0.0294	−0.0160	0.0732		0.0322	−0.0218	−0.0572	0.0367	0.0104

^a^Raw gap in HAZ scores computed as rural HAZ scores- urban HAZ scores. The negative sign of the observed raw gap figures reflects the fact that rural HAZ scores are lower than urban HAZ scores.

^b^Figures in brackets show percentage contribution of individual characteristics to the total explained effect.

Significant effects are in bold. Asterisks denote level of significance - ***, ** and * denote significance at the 1%, 5% and 10% level of significance respectively.

Figures 1 and 2 and Tables 4 and 5 show that in both Bangladesh and Nepal, differences between rural and urban HAZ scores are quite similar across quantiles. In both countries, the ‘counterfactual’ HAZ distribution curves nearly coincide with the urban HAZ distribution, particularly in the lower half of the distribution, suggesting that covariate differences explain the bulk of the rural-urban gap in the distribution of HAZ scores. That differences in socio-economic characteristics (covariate effects) account for a dominant share of rural-urban differences is confirmed by the information presented in the Tables 4 and 5. In Bangladesh, the covariate effect accounts for 62%-95% of the overall differences in HAZ scores in different quantiles, while in Nepal the share of the covariate effect ranges from 72%-97%. The covariate effect is also stronger in the lower quantiles. The co-efficient effect accounts for 5%-37% of the overall differences in HAZ scores in Bangladesh and is more pronounced only in the higher quantiles, while in Nepal the coefficient effect accounts for 3%-28% of the overall differences in HAZ score, with the largest contribution in the median quantile.

The further decomposition of the covariate and co-efficient effects into the contribution of individual covariates in Bangladesh and Nepal is also presented in Tables 4 and 5 respectively. This decomposition shows the relative contribution of individual covariates to child nutrition outcomes in rural and urban areas in the two countries and how they vary across quantiles. The negative sign of the observed raw gap in HAZ scores between rural and urban areas reflects the fact that rural HAZ scores are lower than urban HAZ scores in all quantiles. This must be kept in mind while interpreting the direction of effect of the contribution of individual characteristics in the lower part of Tables 4 and 5 – negative figures imply a contribution to *increasing* the rural-urban disparity in HAZ scores, while positive figures show a contribution to *reducing* it. A large proportion of the covariate effect is accounted for by a limited number of characteristics. In Bangladesh, in the lowest quantile (Q10), wealth (39%), mother’s education (26%) and spouse’s education (22%) account for 87% of the covariate effect which is explained by socio-economic characteristics included in the model. The contribution of other characteristics like child vaccination, working status of the mother, number of children below five years, dependency ratio and living in extended families is relatively small and is significant only in some quantiles. As we move from the lower to the higher quantiles, the contribution of mother’s education decreases while that of wealth increases. The contribution of spouse’s education to the covariate effect is nearly 20% across all quantiles. In Nepal, in the lowest quantile, wealth (38%), mother’s education (22%) and spouse’s education (22%) account for nearly 72% of the covariate effect. The contributions of child vaccination and the working status of the mother are significant in all quantiles, while the dependency ratio is significant in some of the higher quantiles. However, in Nepal, the contribution of mother’s education increases as we move up the quantiles, while that of wealth decreases. The contribution of spouse’s education is much lower than in Bangladesh.

Tables 4 and 5 show that the co-efficient effect in both Bangladesh and Nepal is predominantly due to the differential effects of wealth in rural and urban settings. A unit increase in the wealth index has a stronger association with child HAZ scores in urban households than in rural households. The co-efficient effect of the wealth index tends to widen rural-urban disparities, but in Bangladesh the disadvantage of rural areas is partially offset by the positive effect of extended families on child nutrition in some quantiles.

## Discussion

The QR-based decomposition methods provide specific insight into the drivers of disparities in the lowest quantiles of HAZ scores, which is useful for designing interventions aimed at vulnerable households with the highest levels of stunting. The quantification of the contribution of individual socio-economic determinants to rural-urban disparities can be used to assess the “returns” to different types of interventions. In both countries, rural-urban gaps in the lower half of the distribution are largely accounted for by differing levels of covariates, suggesting that bridging rural-urban inequality in undernutrition is largely a matter of equalizing endowments of the determinants of nutrition. Our results also suggest that much of this can be achieved by focussing on just three determinants: maternal education, spouse’s education and the wealth index. Other variables, child vaccination, age of mother, mother’s working status, extended family dummy and the dependency ratio make a relatively small contribution to explaining rural-urban disparities, especially in the lower quantiles*.* We discuss below the implications for the design of policies and programmes in public health and complementary areas.

The preeminent role of maternal education in child nutrition in the region is emphasised by our results. The contribution of improved maternal education to bridging rural-urban disparities is the largest in the lowest quantiles of HAZ scores and is comparable in magnitude to the contribution of the wealth index (improvements in economic status) Plugging the maternal education gap is, therefore, particularly important for alleviating rural-urban disparity at the lower tail of HAZ. Bangladesh has already made great strides in improving rural women’s education over the last two decades. The Female Secondary Stipend (FSS) programme, a conditional cash transfer programme introduced in 1994, provided impetus for a rapid and substantial expansion in female secondary school enrolment that saw enrolment proportion increase from 35% to more than 50% within a decade [38]. The FFS was implemented only in rural and non-metropolitan urban areas, and is thus likely to have served to equalise the endowment of mother’s education across rural and urban areas. However, as Table 1 indicates, as of 2006 there was still a gap of 1.6 mean years of mother’s education across rural (4.3 years) and urban (5.9 years) areas of Bangladesh in our sample. Thus continued efforts to bridge this gap will likely also continue to pay dividends in terms of reducing rural-urban nutritional inequality. The scope for attaining this improved nutritional equality dividend is altogether larger in Nepal, where women’s educational outcomes remain worryingly poor, particularly in rural areas. This is reflected in the gap of 2.2 mean years across urban (4.2 years) and rural (2 years) areas in our sample. As in many other spheres, education policymaking in Nepal is likely to have been severely hampered by decades of conflict and state fragility. In the current, more stable environment, Bangladesh’s conditional cash-transfer based model to boost women’s education may serve as a useful model for Nepalese policymakers and development agencies.

An important caveat to bear in mind regarding public policymaking in the education sphere is the potential for gender-targeted education programmes to encourage the development of reverse gender-gaps. There is evidence to show that boys’ enrolment in co-educational schools in Bangladesh has been falling relative to girls [39], and that an intra-household reverse gender gap has opened up that could be associated with the FFS [38]. Our results indicate that the education of both girls as well as boys is important for reducing rural-urban nutritional inequality. From this perspective, while it is still critically important to further boost the education of girls, it is also important for policies and programmes to think carefully about how unintended consequences in terms of discouragement of the education of boys is avoided.

Bridging the gap in spouse’s education is also important for reducing rural-urban inequality across the HAZ distribution in Bangladesh, and for the most nutritionally vulnerable children in Nepal. In Bangladesh our results are consistent with a scenario wherein investments in improving the education level of spouses (in addition to investments in maternal education) have large impacts on child nutrition outcomes across the distribution of HAZ scores. This probably highlights the role of the spouse in a context where women may be constrained by social norms in accessing public health messages or services. However, in Nepal, investments in improving the education levels of spouses may have large impact only in the lower end of the HAZ score distribution. Similarly, measures to mitigate the adverse effects of mothers’ working status in rural areas can be expected to have a substantial impact on reducing rural-urban disparities in Nepal. But in Bangladesh, such measures are likely to have only a very limited impact. The decomposition exercise can, therefore, provide useful inputs for decisions on the relative priorities for different types of interventions in specific contexts.

The difference in relative endowments of wealth is the single most important factor in explaining rural-urban disparities in our case study countries. Given the wealth index is a composite of several variables, as discussed before, it is difficult to interpret wealth effects in terms of specific interventions or policies. However, we note that sanitation (toilet facilities) and water (source of supply) are two of the key components of the wealth index in the two countries. Recent research in the region [40] has highlighted the particular importance of sanitation for child nutrition in areas of high population density. As in the case of education, while both countries can reduce rural-urban nutrition inequality by bridging gaps in improved sanitation and water supply, the potential for doing so is larger in Nepal than in Bangladesh. 27% of households in the urban sample and 59% of households in the rural sample in Bangladesh still have access to only ‘unimproved’ toilet facilities. The corresponding numbers for Nepal are 45% for urban and 79% for rural households. In Bangladesh, less than 1% of the urban sample and only 4% of the rural sample use water from unimproved sources. In Nepal, the corresponding numbers are 9% for the urban sample and 22% of the rural sample. Bangladesh’s success in providing safe drinking water over the last few decades has been facilitated by the promotion of cheap shallow hand-pump technology.

The *quality* of rural services in the region relevant to nutrition, such as in health care, have been called into question [9,10]. The co-efficient effects of the core socio-economic determinants are however, relatively very small in magnitude. An important implication for public health programmes arising from our results is that there is no evidence that there is a substantial rural-urban service quality differential impinging on rural-urban child nutrition gap. For example, our results show that mother’s education, measured in years, is an important determinant of the rural-urban nutrition gap, and that this is largely a covariate, rather than a coefficient, effect. If rural education were of inferior quality to urban education, the strength of association between years of education and nutrition is likely to be weaker in rural areas compared to urban, and the coefficient effect would be larger. Also, maternal education impacts child nutrition partly through the use of more proximal determinants like diets and use of health care and ante/post-natal facilities [2]. The lack of importance of coefficient effects implies that the limiting constraint in rural-urban inequality is not the relative quality of food availability or health services in these areas, but rather the education endowments required to utilize them. Thus interventions that close these endowment gaps will effectively lower nutrition inequality. Note that this is not to say that service quality issues are not important to child nutrition; rather, the quality differentials are not currently large enough to be driving rural-urban nutrition inequality.

### Limitations of the study

We have used QR-CD methods to assess the relative importance of covariate and coefficient effects in explaining rural-urban disparities in child nutrition using cross-sectional data sets for two countries that are at different stages of development and urbanisation. The efficacy of socio-economic endowments and public health infrastructure in promoting improved child nutrition may change as a country develops. QR-CD methods can provide more insights into the changes in the efficacy of public health interventions if they can be applied to repeated cross-section datasets within a country. This is an extension of the study that we intend to explore subject to the availability of data. It must be noted that QR-CD methods are computationally intensive and require large datasets. The method cannot be applied to smaller datasets such as those available from intervention studies or RCTs. The CD exercise can provide reliable results only if the basic quantile regression includes all the important determinants of child nutrition outcomes and is well specified. While our choice of determinants has been constrained by the coverage of the DHS surveys, we have included the key exogenous demographic and economic determinants considered in the previous literature [2,11,13]. The linear specification adopted for the quantile regression may not accommodate non-linear and threshold effects associated with the determinants. We have, however, tested for such effects for key variables like maternal education and spouse’s education.

## Conclusions

Using DHS datasets we examined rural-urban differences in child nutrition outcomes using HAZ scores for two South Asian countries – Bangladesh and Nepal which differ substantially in the extent of urbanisation. The similarity in the pattern of rural-urban differentials in these two countries suggests that these differentials persist even as urbanisation and economic development proceed. The methodology employed in this paper allows us to decompose rural-urban differences in child nutrition outcomes into covariate and co-efficient effects and further enables us to quantify the contribution of individual explanatory variables (socio-economic characteristics) to rural-urban differences via these effects. The decomposition of rural-urban differences into covariate and co-efficient effects shows that the covariate effect is dominant. A core set of determinants – wealth index (which incorporates ownership of assets and access to sanitation and drinking water), maternal education and spouse’s education – accounts for a very large proportion of the covariate effects in both countries, which suggests that there are no fundamental differences in the socio-economic determinants of child nutrition outcomes in rural and urban areas. The dominance of covariate effects confirms findings from earlier studies [2,11] that rural-urban disparities in child nutrition are primarily attributable to the difference in levels of critical determinants and that differences in the strength of association between determinants and nutrition outcomes are of relatively small magnitude. Our analysis suggests that public health interventions aimed at overcoming rural-urban disparities in child nutrition outcomes need to focus principally on bridging gaps in socio-economic endowments and improving the quality of rural infrastructure. The improvement of child nutrition outcomes in developing countries does not appear to call for fundamentally different approaches to public health interventions in rural and urban areas.

## Endnotes

^a^ The DHS surveys generally cover water supply, electricity, sanitation, flooring type, ownership of assets such as radio, television, telephone, and refrigerator, ownership of agricultural land and livestock, persons sleeping per room and country specific items. Ownership of agricultural land and livestock is not used in the calculation of the DHS Wealth Index as these assets are not generally available in urban areas.

^b^ There have been some concerns that the DHS Wealth Index is too “urban” in its construction depending on assets and services that the urban population may have but the rural population may not have. This has been sought to be addressed in more recent DHS surveys by inclusion of more items mainly rural in character (e.g., water pumps, grain grinders). A number of alternative approaches to construction of rural and urban wealth indices using DHS data are possible [41] including construction of separate rural and urban indices. Separate indices for rural and urban areas would not be useful for our purposes as the methods used in this study require that the determinants of HAZ scores are measured in the same way in rural and urban areas.

## Competing interests

The authors have no competing interests to declare.

## Authors’ contributions

CSS contributed to the literature review, design of the study, interpretation of results and prepared the drafts of the manuscript. GZ managed the DHS datasets, ran the econometric models for unconditional quantile regressions and counterfactual decompositions and contributed to the interpretation of results. BS conceived of the study, participated in its design, provided oversight and co-ordination and helped in revisions of the manuscript. All authors read and approved the final manuscript.

## Authors’ information

Dr C.S. Srinivasan is a Senior Lecturer in the Department of Food Economics and Marketing, School of Agriculture, Policy and Development at the University of Reading, UK. Dr Giacomo Zanello is a Research Officer in the Oxford Department of International Development, Queen Elizabeth House, University of Oxford, UK. Professor Bhavani Shankar is Professor of International Agriculture, Food and Health in the Centre for Development, Environment and Policy in the School of Oriental and African Studies, University of London and the Leverhulme Centre for Integrative Research on Agriculture and Health.

## Pre-publication history

The pre-publication history for this paper can be accessed here:

http://www.biomedcentral.com/1471-2458/13/581/prepub

## Supplementary Material

Additional file 1 Counterfactual Decomposition Procedure Using Unconditional Recentred Influence Function (RIF) Quantile Regression. Click here for file
