# Supplementary material for: Rural-urban disparities in child nutrition in Bangladesh and Nepal
Source: BMC Public Health. 2013 Jun 14;13:581. doi: 10.1186/1471-2458-13-581 (PMC3729423; doi:10.1186/1471-2458-13-581)
Supplement: Additional file 1 — Counterfactual Decomposition Procedure Using Unconditional Recentred Influence Function (RIF) Quantile Regression. [file 1471-2458-13-581-S1.docx]

**Appendix-1: Counterfactual Decomposition Procedure Using Unconditional Recentred Influence Function (RIF) quantile regression**

To assess the rural-urban differentials in HAZ scores, we first estimate the distributions of HAZ scores separately for rural and urban children in each country using kernel smoothing techniques. From the kernel density estimates of HAZ scores, the rural-urban differential is computed at each quantile and provides the raw difference in HAZ scores across the distribution.

Following Firpo et al. [1], the decomposition of differences between rural and urban HAZ scores (for each country) proceeds in two steps. In the first step, a counterfactual distribution of rural HAZ scores is constructed using a reweighting procedure suggested by Di Nardo et al. [2]; this is the distribution of HAZ scores in rural areas that would have prevailed if rural households had the same returns to their characteristics as the urban population. If *q (HAZ_R_)* and *q (HAZ_U_)* are given quantiles of the HAZ score distribution in urban and rural areas, and *q (HAZ_C_)* is the same quantile of the counterfactual rural distribution, then the overall difference between rural and urban HAZ scores at any given quantile can be decomposed as:

*q (HAZ_R_)* - *q (HAZ_U_)* = [*q (HAZ_R_)* - *q (HAZ_C_)*] + [*q (HAZ_C_)* - *q (HAZ_U_)*] (1)

where [*q (HAZ_R_)* - *q (HAZ_C_)*] represents the covariate effect and [*q (HAZ_C_)* - *q (HAZ_U_)*] represents the coefficient effect. In the second step, the covariate and coefficient effects are each decomposed into the contribution of individual covariates using the Recentred Influence Function (RIF) regression [3] to obtain unconditional quantile effects of covariates on HAZ scores. The RIF regression is of the form:

*E (RIF (HAZ|q_τ_) = Xβ* (2)

where β represents the unconditional effect of covariate X on quantile τ of HAZ scores. The RIF unconditional regressions are separately estimated for rural, urban and counterfactual rural HAZ score distributions:

$\hat{RIF}\left( {HAZ}_{K},\hat{q_{\tau}} \right)= X_{K}\hat{\beta_{K}}$ (3)

and R represents rural, U represents urban and C represents the rural counterfactual. Using the RIF unconditional quantile estimates the following decomposition can be obtained for any given quantile:

$\hat{q_{\tau}}\left( {HAZ}_{R} \right)- \hat{q_{\tau}}\left( {HAZ}_{U} \right)= \left[ \bar{X_{R}}\left( \hat{\beta_{C}} \right.-\left. \hat{\beta_{R}} \right)+ \hat{R^{Coeff}} \right]+\left[ \left( \bar{X_{U.}}\hat{\beta_{U}}-\bar{X_{R.}}\hat{\beta_{C}} \right)+\hat{R^{Cov}} \right]$ (4)

where $\hat{q_{\tau}}\left( {HAZ}_{R} \right)- \hat{q_{\tau}}\left( {HAZ}_{U} \right)$represents the raw difference in rural and urban HAZ scores at the τ^th^ quantile and X represents the covariate averages. Note that $\hat{\beta_{C}}$is estimated from an RIF regression of the counterfactual HAZ score distribution. $\left( \hat{\beta_{C}} \right.-\left. \hat{\beta_{R}} \right)$ is, therefore, the difference in the effects of covariates between rural and urban areas and $\bar{X_{R}}\left( \hat{\beta_{C}} \right.-\left. \hat{\beta_{R}} \right)$represents the coefficient effect. $\left( \bar{X_{U.}}\hat{\beta_{U}}-\bar{X_{R.}}\hat{\beta_{C}} \right)$represents the differences between rural and urban HAZ scores attributable to the differences in characteristics of endowments and hence represents the covariate effect. $\hat{R^{Coeff}}$and $\hat{R^{Cov}}$are errors related to the estimation of coefficient and covariate effects.

**References**

| 1. | Firpo S, Fortin NM, Lemieux T: **Unconditional quantile regressions**. *Econometrica* 2009, **77**(3):953-973. |
| --- | --- |
| 2. | DiNardo J, Fortin NM, Lemieux T: **Labor Market Institutions and the Distribution of Wages, 1973-1992: A Semiparametric Approach**. *Econometrica* 1996, **64**(5):1001-1044. |
| 3. | Firpo S, Fortin N, Lemieux T: **Decomposing wage distributions using recentered influence function regressions**. Working Paper, *University of British Columbia (June)* 2007. |
